# Supplementary material for: Genetic regulation of spermine oxidase activity and cancer risk: a Mendelian randomization study
Source: Sci Rep. 2021 Aug 31;11:17463. doi: 10.1038/s41598-021-97069-x (PMC8408253; doi:10.1038/s41598-021-97069-x)

**Figure S1.** Association between rs1741315 alleles and *SMOX* RNA-seq expression (508 adults; EGCUT cohort).

**
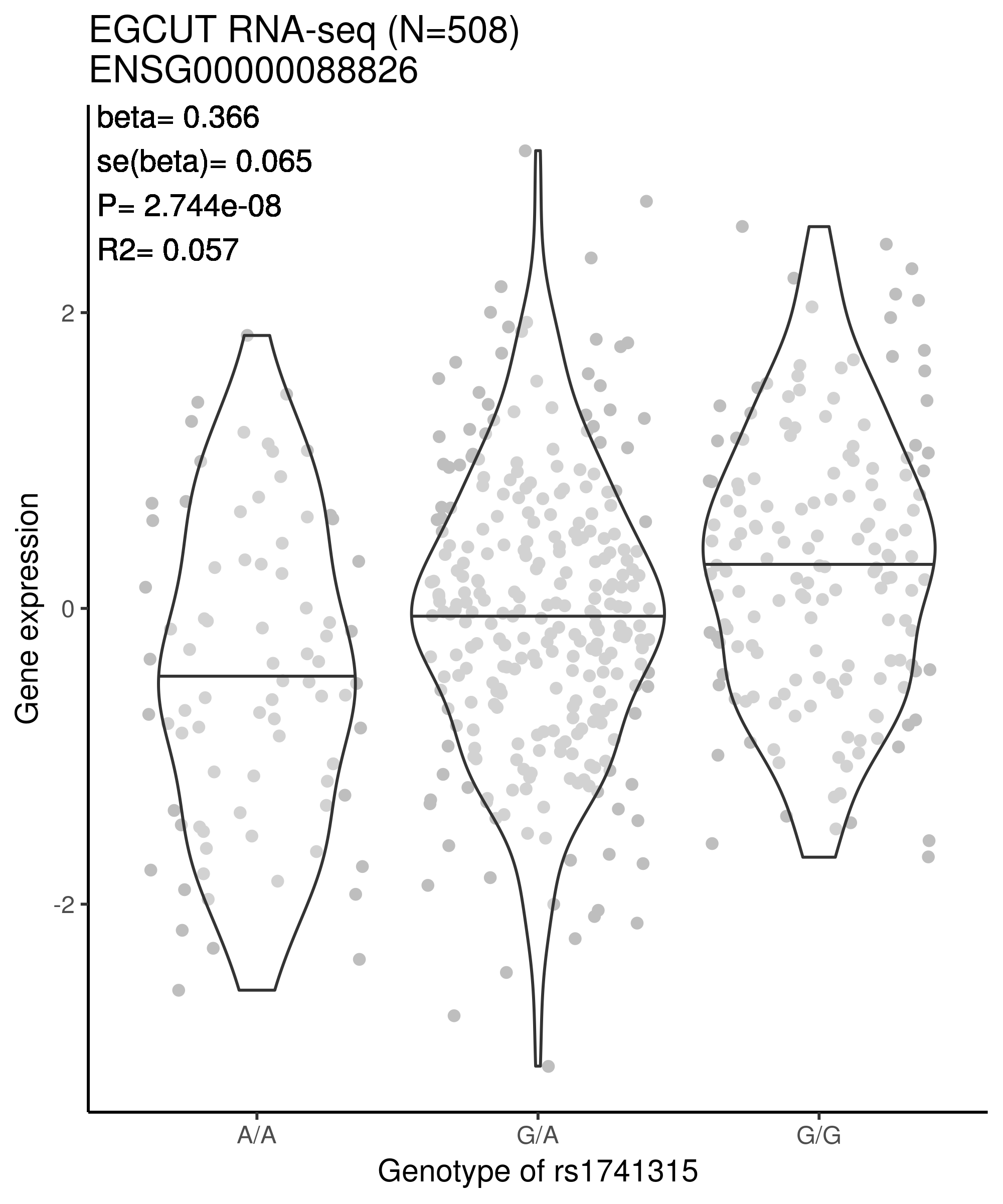
**

**Figure S2.** Association between rs1741315 alleles and *SMOX* RNA-seq expression, stratified into 10-year age groups (494 adults; EGCUT cohort).

**
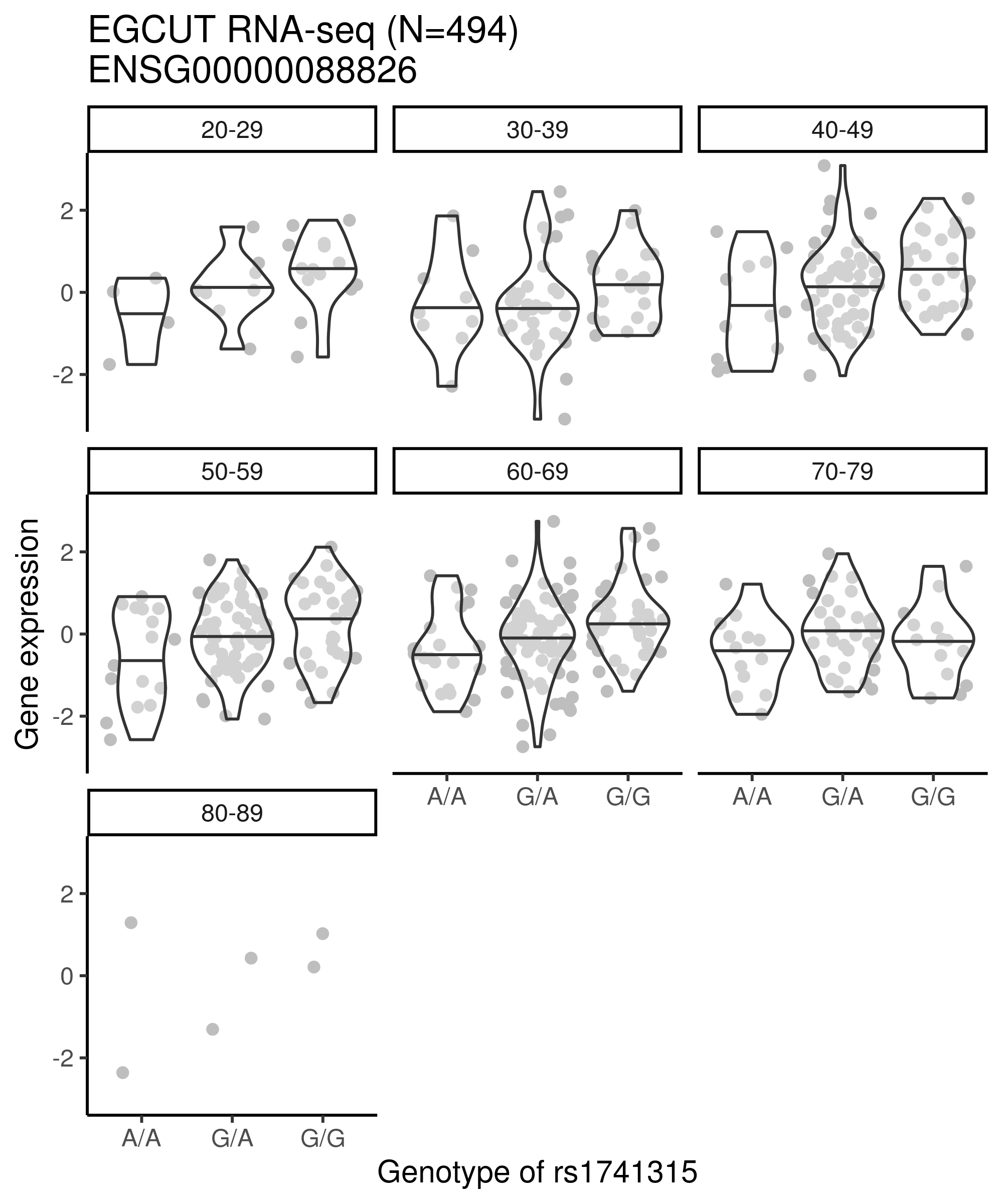
**

**Figure S3.** Interaction between donor age at blood draw and rs1741315 genotype on *SMOX* expression (494 adults; EGCUT cohort).

**
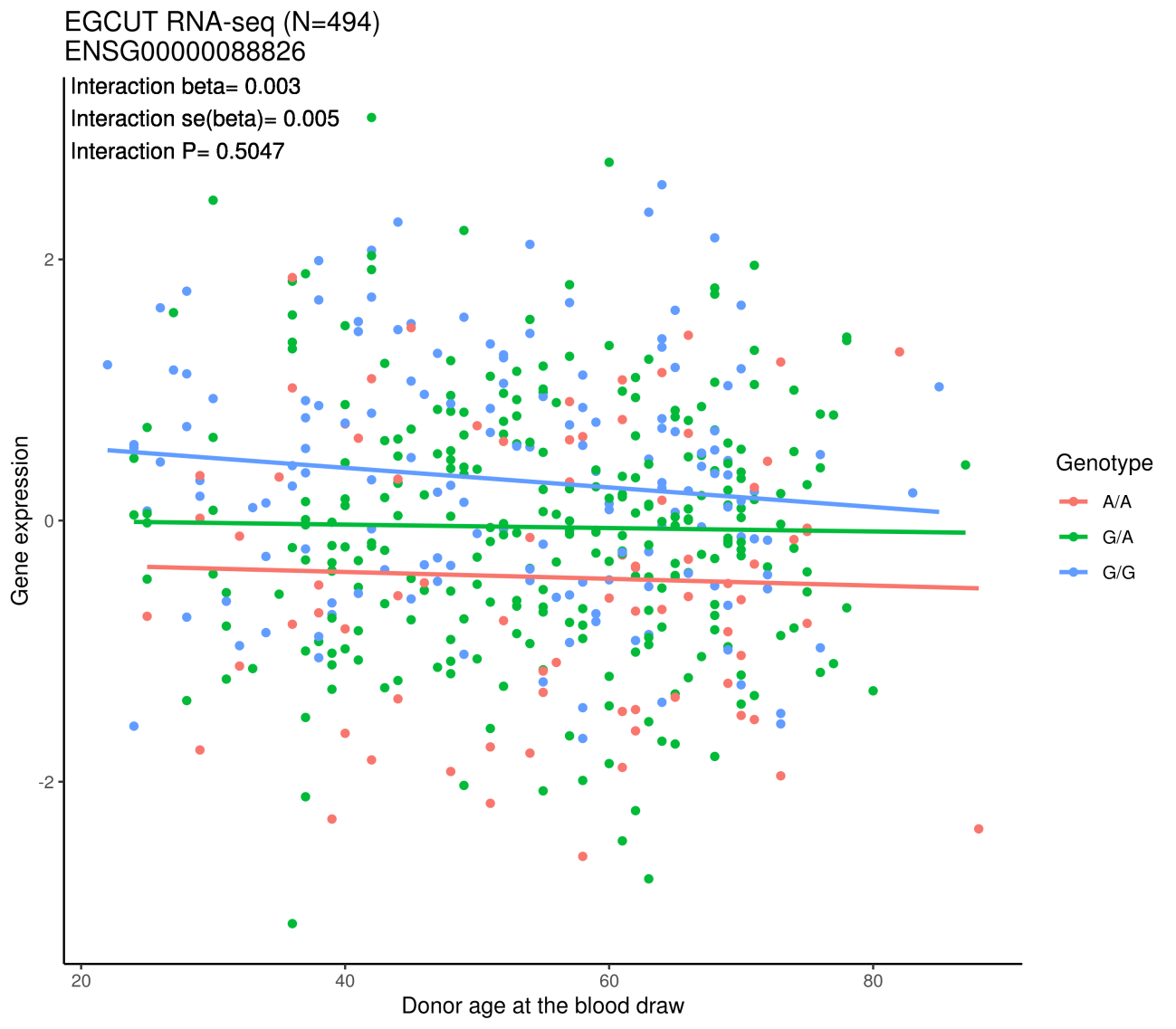
**

**Figure S4.** Association between methylation proportion of the CpG cg07472708 and rs1741315 (305 adults; EGCUT cohort).

**Figure S5.** Association between methylation proportion of the CpG cg07472708 and rs1741315, stratified into 10-year age groups (305 adults; EGCUT cohort).

**Figure S6.** Interaction between donor age at blood draw and rs1741315 genotype on cg07472708 methylation levels (305 adults; EGCUT cohort).

**Figure S7.** Boxplot of the rs1741315 alleles (0=GG; 1=AG; 2=AA) versus spermidine/spermine ratio, stratified by IHPS disease status (534 samples; SSI-IHPS cohort). 1= control; 2 = IHPS case.


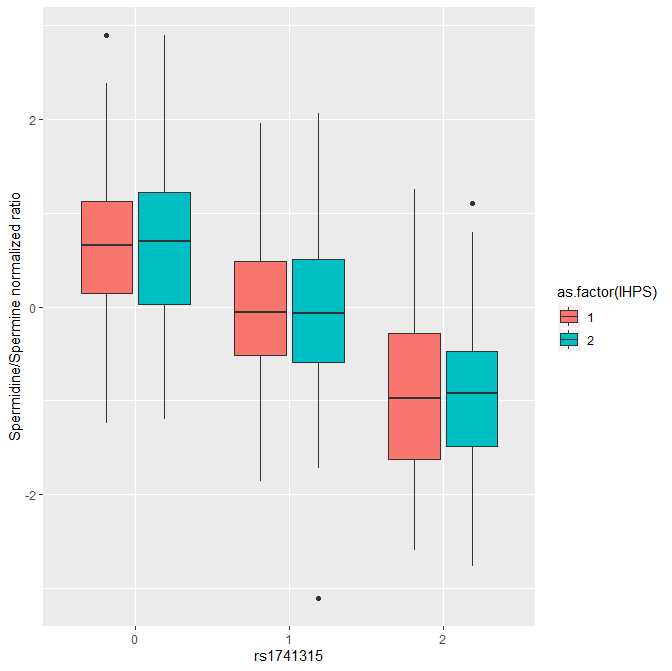


**Figure S8.** Boxplot of the rs1741315 alleles (0=GG; 1=AG; 2=AA) versus spermidine/spermine ratio, stratified by sex (534 samples; SSI-IHPS cohort). 1= boys; 2 = girls.


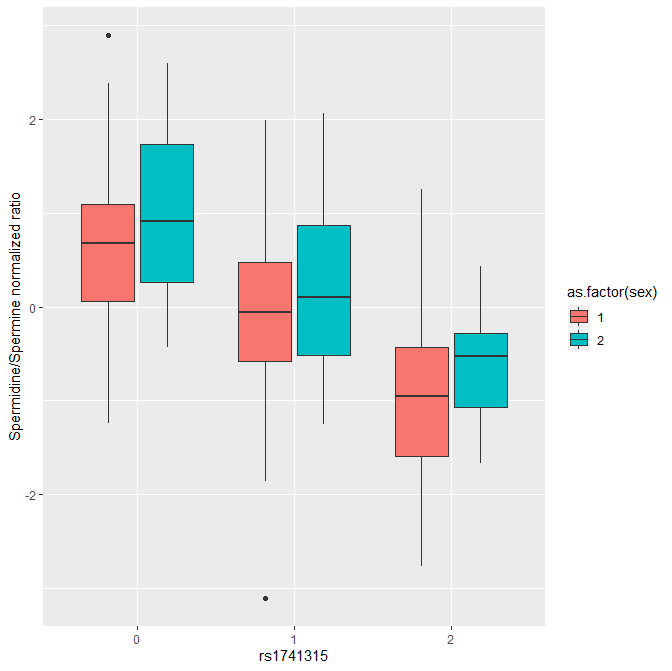


**Figure S9.** Boxplot of the rs1741315 alleles versus spermidine/spermine ratio (534 samples; SSI-IHPS cohort).

**
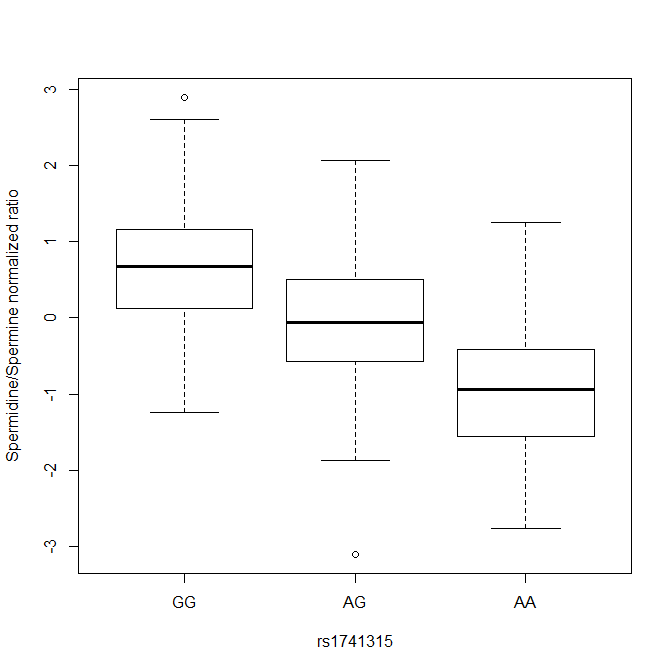
**

**Figure S10.** Density distribution of Spermidine normalized concentration (μM) (534 samples; SSI-IHPS cohort).


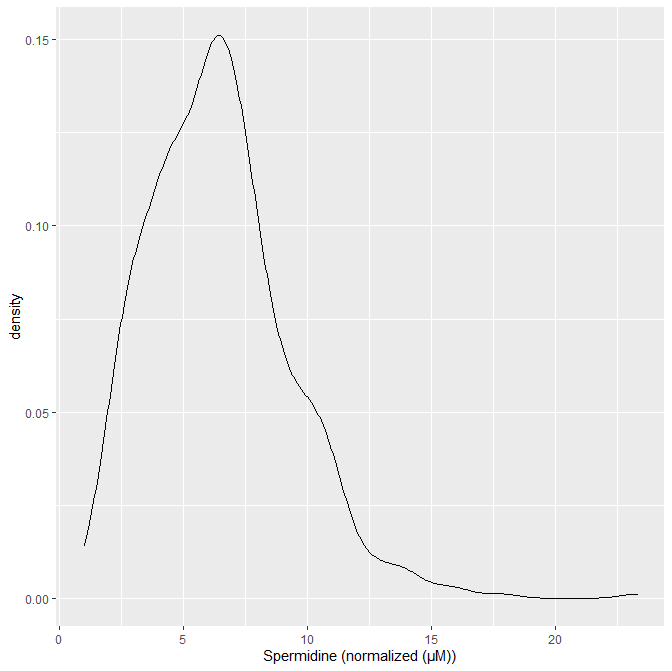


**Figure S11.** Density distribution of Spermine normalized concentration (μM) (534 samples; SSI-IHPS cohort).


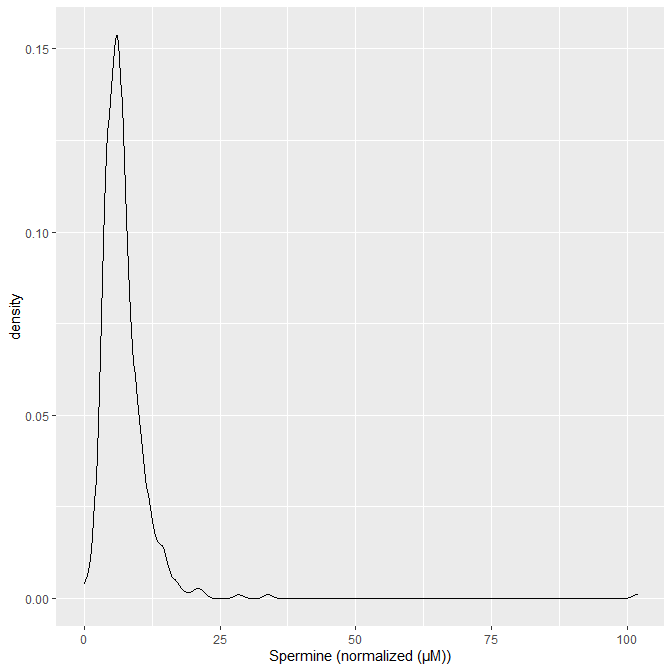


**Figure S12.** Density distribution of inverse normally transformed (Spermidine/Spermine) (534 samples; SSI-IHPS cohort).


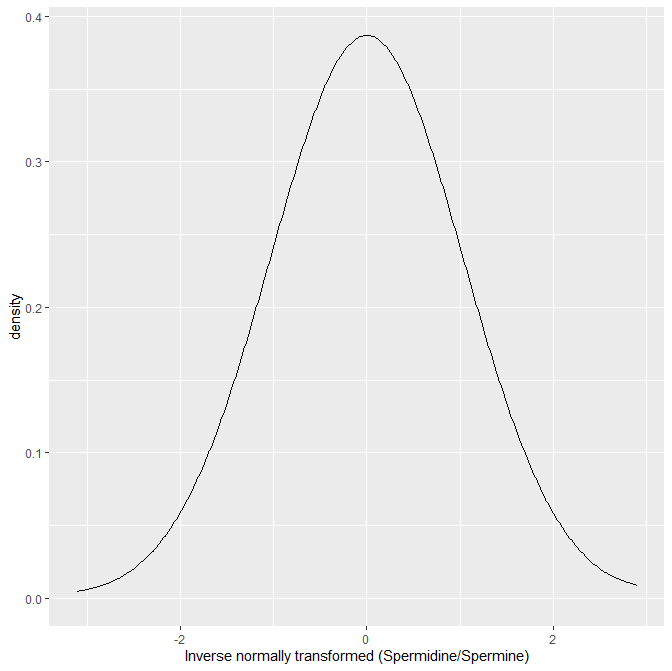

Supplement: Supplementary file 1 — Supplementary Information 1. [file 41598_2021_97069_MOESM1_ESM.docx]
